# Artefacts in Cone Beam CT Mimicking an Extrapalatal Canal of Root-Filled Maxillary Molar

**DOI:** 10.1155/2013/797286

**Published:** 2013-03-31

**Authors:** Carla Cristina Camilo, Manoel Brito-Júnior, André Luis Faria-e-Silva, Alex Carvalho Quintino, Adrianne Freire de Paula, Antônio Miranda Cruz-Filho, Manoel Damião Sousa-Neto

**Affiliations:** ^1^Department of Dentistry, State University of Montes Claros, Avenue Rui Braga s/n, 39401-089 Montes Claros, MG, Brazil; ^2^Department of Dentistry, School of Dentistry, Federal University of Sergipe, Rua Cláudio Batista s/n, 49060-100 Aracaju, SE, Brazil; ^3^Department of Restorative Dentistry, Faculty of Dentistry, University of São Paulo, Avenue Bandeirantes 3900, 14040-900 Ribeirão Preto, SP, Brazil

## Abstract

Despite the advantages of cone-beam computed tomography (CBCT), the images provided by this diagnostic tool can produce artifacts and compromise accurate diagnostic assessment. This paper describes an endodontic treatment of a maxillary molar where CBCT images suggested the presence of a nonexistent third root canal in the palatal root. An endodontic treatment was performed in a first maxillary molar with palatal canals, and the tooth was restored with a cast metal crown. The patient returned four years later presenting with a discomfort in chewing, which was reduced after occlusal adjustment. CBCT was prescribed to verify additional diagnostic information. Axial scans on coronal, middle, and apical palatal root sections showed images similar to a third root canal. However, sagittal scans demonstrated that these images were artifacts caused by root canal fillings. A careful interpretation of CBCT images in root-filled teeth must be done to avoid mistakes in treatment.

## 1. Introduction

Knowledge about the anatomy of the root canal system is essential for successful endodontic therapy and permits a correct diagnosis followed by thorough cleaning and shaping of root canals [1, 2]. An accurate observation of internal anatomy is important mainly in teeth presenting complex and variable configurations related to root canals, such as maxillary molars. Unfortunately, these teeth present important variations in number of roots and root canals. The presence of three roots (two buccal and one palatal) and three root canals is a frequent condition for this tooth. It is not uncommon for there to be a second root canal in the mesiobuccal root, increasing the total of root canals to four [2]; however, the presence of an additional root canal has been reported for mesiobuccal, distobuccal, and palatal roots [3–6]. These anatomical variations can compromise the endodontic therapy when not observed.

Traditionally, radiographs are used to assess the anatomic aspect of root canals, enabling verification of the number and location of root canals and the presence of curvatures that could hinder the instrumentation [7]. However, the correct visualization of the root canal system can be jeopardized by the two-dimensional view of radiographs, while the object evaluated presents three dimensions. Recently, the use of three-dimensional (3D) imaging techniques such as cone beam computed tomography (CBTC) has gained popularity in endodontic therapy [8].

The 3D images provided by CBCT permit the view of greater details of the root canal anatomy than radiographic images [9]. The improved evaluation of the internal anatomy of root canal systems is an important diagnostic tool for the proper therapy of all root canals in morphologically complex teeth [10, 11]. Despite these advantageous, however, artifacts can be produced in the CBCT images that may lead to a misdiagnosis related to the number and localization of root canals [12]. In this situation, an accurate clinical diagnosis assumes crucial importance to the prevention of unwanted procedure errors. 

This paper describes the endodontic management of a first maxillary molar for which artifacts in a CBCT follow-up exam mimicked an extra palatal canal. 

## 2. Case Presentation

**Figure 1:**
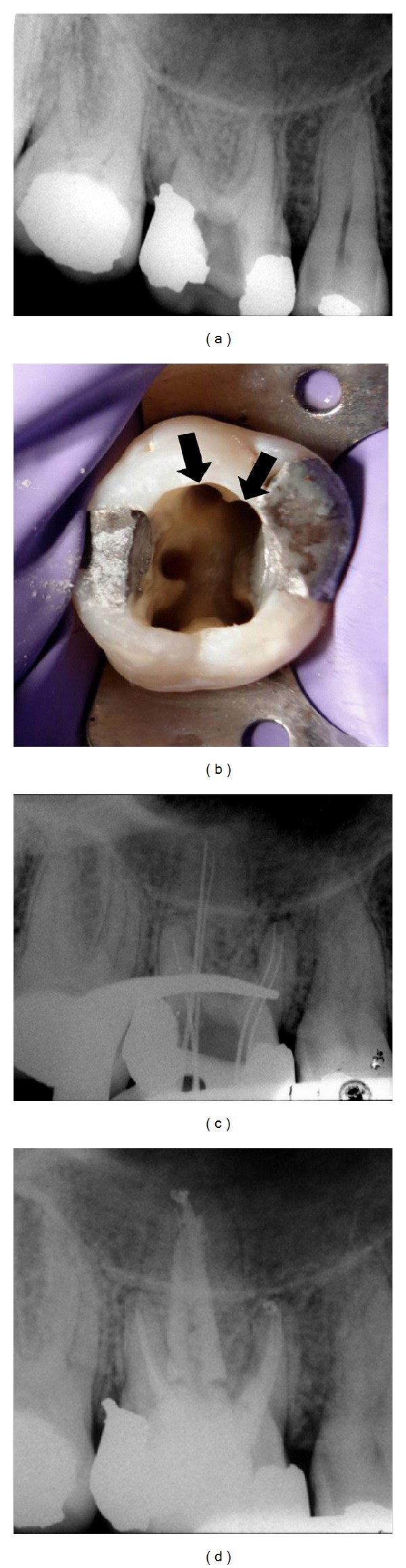
(a) Initial radiographic aspect of tooth number 3. (b) Endodontic access showing the presence of five root canal entrances. (c) Radiograph for working length determination. (d) Radiographic aspect after root canal fillings.

A 27-year-old man was referred for endodontic treatment of tooth number 3. The patient related episodes of spontaneous pain in the previous week, while an emergency procedure was performed. Clinical examination revealed that the pulp chamber was exposed, and no response to thermal pulp testing (Endo-Ice, Maquira Indústria de Produtos Odontológicos Ltda, Maringá, PR, Brazil) was observed. Slight responses to palpation and percussion were verified. A periapical radiograph showed normality of the periapical region (Figure 1(a)). The diagnosis of pulp necrosis was established and the endodontic treatment initiated.

Under local anesthesia and rubber dam, the coronal access was performed and the root canals' entrance was explored using an endodontic explorer. Identified were the two mesiobuccal canals, the distobuccal canal, and, unexpectedly, two orifices in the palatal root, corresponding to mesio- and distopalatine canals (Figure 1(b)). After the root canals instrumentation, the radiograph taken for working length determination confirmed the presence of five root canals (Figure 1(c)). Chemomechanical preparation was performed by using ProTaper NiTi rotatory instruments (Dentsply-Maillefer, Ballaigues, Switzerland) and irrigation with 2.5% sodium hypochlorite. A calcium-hydroxide-based paste (Calen, SS White, Rio de Janeiro, RJ, Brazil) was used as intracanal dressing and the provisional restoration was performed with a zinc oxide and eugenol restorative material (IRM, Dentsply, Petrópolis, RJ, Brazil).

The patient returned for a second appointment after eight days. The temporary restoration was removed under local anesthesia and rubber dam. The intracanal dressing was removed, and the root canals were dried and filled with gutta-percha cones (Konne, Belo Horizonte, MG, Brazil) and sealer (Endofill, Dentsply, Petropolis, RJ, Brazil) by lateral condensation technique. Afterwards, the tooth was restored with composite resin. 

Four years later, the patient returned to the clinic complaining of discomfort when chewing on tooth number 3, which had received a cast metal restoration approximately six months earlier. Clinical examination showed that the tooth had no tenderness on palpation and no mobility. The mean probing pocket depth was within normal limits. Slight pain to percussion was observed and the radiograph showed absence of periapical lesion. Upon checking the occlusion, a premature contact was detected on the metal restoration of tooth number 3. Occlusal adjustment was performed, resulting in a gradual resolution of the symptoms. 

**Figure 2:**
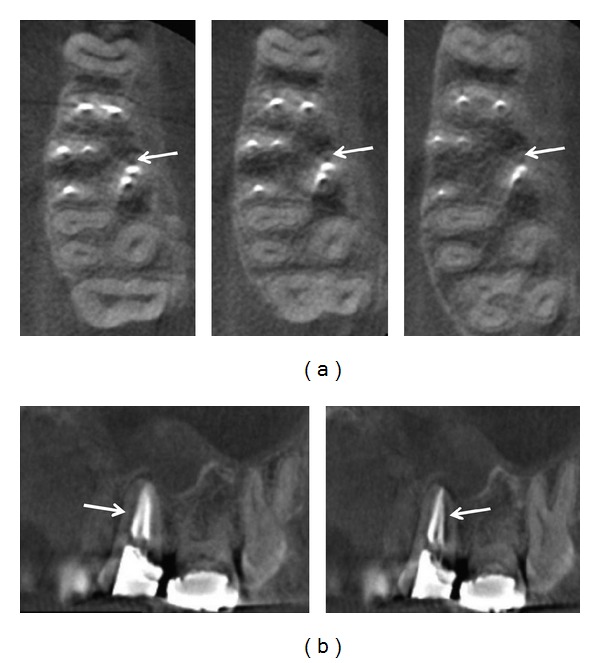
(a) From left to right, axial scans of coronal, middle, and apical root sections suggesting the presence of a third root canal in the palatal root (arrows). (b) Sagittal scans demonstrating that the third canal observed at previous images was an artifact (arrows). Note the presence of hypodense periapical areas on the palatal root.

Despite this, the patient was advised that CBCT examination could be useful in providing additional information on the case. Thus, a small-volume CBCT scan (Kodak 9000 3D, Carestream Health, Inc., Rochester, NY, USA) of tooth number 3 with exposure parameters of 70 kV, 5.0 mA, and 0.076 mm (isotropic voxel) was obtained after informed consent from the patient. An image suggesting the presence of a third root canal in the palatal root was observed in the axial scans (Figure 2(a)); however, sagittal scans demonstrated that the previous images were artifacts caused by root canal fillings (Figure 2(b)). Small hypodense periapical areas also were observed in the sagittal views (Figure 2(b)). These images were possibly related to the occlusal trauma or to a healing process of a preoperative lesion not detected radiographically at the initiation of treatment. Thus, it was decided to monitor the case without any additional treatment.

## 3. Discussion 

Additional anatomical information in maxillary molars is provided by CBCT images when compared with limited two-dimensional techniques [13–15]. Despite CBCT not being available during initial treatment in the present case, all root canals were identified and treated based on the operator's knowledge and skills [16]. Careful clinical inspection and suitable access to the cavity were performed, permitting the identification of two-canal orifices on the palatal root, which represents an unusual root canal configuration [3, 4]. CBCT was recommended at the follow-up period, considering the clinical findings and the importance of 3D images to identify untreated root canals [11]. At first, a diagnostic dilemma was established owing to the possible presence of an extra canal in the palatal root observed on CBCT images. However, these images were characterized as artifacts produced by root canal fillings after a detailed CBCT data assessment. 

An understanding of CBCT technology, including its properties and limitations, is essential to prevent misleading findings. CBCT was introduced into dentistry in the late 1990s, and it is characterized as an imaging modality under constant improvement [8]. High-quality bony definition, low radiation dose, and compact design are advantages of CBCT scans over conventional computed tomography [8, 17]. In addition, CBCT allows a faster image reconstruction in the axial, sagittal, and coronal planes, providing 3D views for diagnosing and managing endodontic complications [9].

One disadvantage of CBCT is the scattered radiation that produces streaking image artifacts, which can prevent diagnostic accuracy. This problem generally occurs during data acquisition after lower jaw movement (motion artifacts) or when a metal object or a dense filling material exists on the tooth structure (metal artifacts) [18]. Untrue CBCT images were observed near metallic intracanal posts, jeopardizing the diagnosis of root perforations [19] and root fractures [20]. Metal artifacts apparently degrade the quality of CBCT images by increasing background noise with a simultaneous decrease in contrast [18]. These artifacts are caused by the beam-hardening effect (i.e., lower energy photons are absorbed in preference to higher-energy photons).

Scanning and reconstructed parameters similar to those used in this paper resulted in suitable image quality for root canal anatomy assessment [21]. The CBCT device used (Kodak 9000 3D) is categorized as small-volume CBCT, based on its scan field of view (FoV), which covers only few teeth or one jaw [8, 22]. Usually, small FoV provides better image quality with lower effective radiation and fewer artifacts when compared with medium and large FoV devices [22]. However, artifacts still occur using a small FoV, reducing the accuracy, sensitivity, and specificity of the CBCT technique, particularly in root-filled teeth [23. 24]. 

A careful interpretation of CBCT data adjusted to the diagnostic task is necessary to attain the examination goals [23]. Krithikadatta et al. [12] reported contradictory findings between CBCT images and clinical aspects evaluating the root canal anatomy of a mandibular first molar. They found clinically the existence of a single root canal within the mesial root, while CBCT artifacts mimicked an additional untreated canal. This challenging case showed the importance of recognizing imaging artifacts for differential diagnosis of anatomical root canal morphology [12]. In the present case, CBCT scans also suggested an extra canal, but the analysis of the images in different reconstructed planes provided sufficient information to correct diagnosis. 

One major reason to interpret CBCT images with caution in the present case was the uncommon occurrence of three palatal canals in the maxillary molars, something only documented in few reports [24, 25]. On the other hand, the presence of an additional canal could justify the hypodense periapical areas, which would be representative of an apical periodontitis. In this scenario, a nonsurgical endodontic retreatment could be indicated; however, the final diagnosis was made in conjunction with the clinical findings, and the monitoring of the case was chosen. In summary, this case indicates that CBCT images should be interpreted carefully in root-filled teeth, taking into account the possibility of artifacts that mimic the presence of additional canals.
